# Nitric Oxide Isotopic Analyzer Based on a Compact Dual-Modulation Faraday Rotation Spectrometer

**DOI:** 10.3390/s151025992

**Published:** 2015-10-14

**Authors:** Eric Zhang, Stacey Huang, Qixing Ji, Michael Silvernagel, Yin Wang, Bess Ward, Daniel Sigman, Gerard Wysocki

**Affiliations:** 1 Department of Electrical Engineering, Princeton University, Princeton, NJ 08544, USA; E-Mails: ejzhang@princeton.edu (E.Z.); sahuang@princeton.edu (S.H.); 2 Department of Geosciences, Princeton University, Princeton, NJ 08544, USA; E-Mails: qji@princeton.edu (Q.J.); bbw@princeton.edu (B.W.); sigman@princeton.edu (D.S.); 3 Department of Electrical Engineering, University of Notre Dame, Notre Dame, IN 46556, USA; E-Mail: michael.p.silvernagel.1@nd.edu; 4 Now with Healthy Photon, 200 East Guoding Road, Shanghai, China; E-Mail: yin.wang@healthyphoton.com

**Keywords:** Faraday effect, optical sensing and sensors, spectroscopy, nitrogen cycle, nitric oxide, isotopic ratiometry

## Abstract

We have developed a transportable spectroscopic nitrogen isotopic analyzer. The spectrometer is based on dual-modulation Faraday rotation spectroscopy of nitric oxide isotopologues with near shot-noise limited performance and baseline-free operation. Noise analysis indicates minor isotope (^15^NO) detection sensitivity of 0.36 ppbv·Hz^−1/2^, corresponding to noise-equivalent Faraday rotation angle (NEA) of 1.31 × 10^−8^ rad·Hz^−1/2^ and noise-equivalent absorbance (α*L*)_min_ of 6.27 × 10^−8^ Hz^−1/2^. White-noise limited performance at 2.8× the shot-noise limit is observed up to ~1000 s, allowing reliable calibration and sample measurement within the drift-free interval of the spectrometer. Integration with wet-chemistry based on acidic vanadium(III) enables conversion of aqueous nitrate/nitrite samples to gaseous NO for total nitrogen isotope analysis. Isotopic ratiometry is accomplished via time-multiplexed measurements of two NO isotope transitions. For 5 μmol potassium nitrate samples, the instrument consistently yields ratiometric precision below 0.3‰, thus demonstrating potential as an *in situ* diagnostic tool for environmental nitrogen cycle studies.

## 1. Introduction

Ratiometric analysis of nitrogen isotopologues (^14^N, ^15^N) provides unique insight into nitrogen cycling dynamics [1,2,3,4,5] and enables environmental diagnostics by differentiating various natural and anthropogenic chemical processes [6,7]. This bears particular significance in light of recent studies [8,9] indicating comparable rates of anthropogenic and natural nitrogen fixation due to combustion of fossil fuels and fertilizer production via the Haber-Bosch process. Additionally, nitric oxide (NO) is known to play a key physiological role as a signal molecule in the human body [10], and isotopic analysis of nitrogen isotopes (in the form of nitrates/nitrites in blood and urine as well as NO in breath) are utilized in medical diagnostics of physiopathological processes [11,12]. In the case of environmental studies, sub-per mille precision is frequently required [1,4,5] as deviations from natural abundance remain small; in the case of medical analysis, isotopically labeled precursors may be introduced into the patient’s body for metabolic studies and the requirements for isotopic sensitivity can be relaxed [11,13]. Presently, stable-isotope mass-spectrometry is the state-of-art technique for precision isotopic ratiometry [5,6,7,11,12], but the substantial capital investment and maintenance requirements involved prevent nitrogen isotope analysis from being used as a mainstream diagnostic tool. Furthermore, the lack of instrument transportability has eliminated the possibility of *in situ* measurements, thus requiring environmental samples to be shipped to the laboratory, which presents practical limits on sample sizes.

In this paper, we present a transportable analyzer based on dual-modulation Faraday rotation spectroscopy (DM-FRS) of NO [13,14,15] with near-fundamental quantum-noise limited sensitivity. The transportability of the system allows *in situ* measurements and practically eliminates the sample size limitation characteristic in the case of external laboratory analysis. Integration of the spectrometer with a compact wet-chemical vanadium(III) conversion setup [15,16] enables measurement of nitrate/nitrite fluid samples. Sub-per mille isotopic ratiometric precision for micro-mole nitrate samples has been achieved, demonstrating our spectrometer as an enabling technology for *in situ* environmental and medical diagnostics.

## 2. Signal and Noise in DM-FRS: Theoretical Analysis and Practical Limitations

Faraday rotation spectroscopy (FRS) techniques have been widely studied in the past [13,14,15,17,18,19,20,21,22] due to its detection selectivity and immunity to diamagnetic species (e.g., H_2_O, CO_2_), as well as the noise-suppression capabilities that allow near shot-noise detection [13]. In its most general form, FRS exploits the magnetic field induced circular birefringence observed for Zeeman-split transitions of paramagnetic molecules (e.g., NO, O_2_, OH, *etc.*). This results in the rotation of polarization of light as it passes through the sample. The rotation is converted to intensity change by projection onto a polarizer (nominally called the *analyzer*), and the resulting intensity is measured by the photodetector. Conventional techniques generally utilize either: (i) magnetic field modulation (AC-FRS) [18,19] or (ii) laser wavelength modulation (DC-FRS) [17,18,20], where the former eliminates optical etalon effects through sample modulation (enabling baseline-free performance) and the latter provides effective reduction of 1/*f* noise. Both techniques have been extensively studied, though the individual use of either AC-FRS or DC-FRS allows for only mutually exclusive noise reduction techniques. Recent work on hybrid-FRS [20] combined DC-FRS with balanced-detection for etalon cancellation and intensity-noise reduction, enabling measurements near (1.4×) the shot-noise limit. However, the lack of low-noise, high common-mode suppression balanced detectors in the mid-infrared limited the implementation of these techniques in this highly desirable molecular fingerprint spectral region.

**Figure 1:**
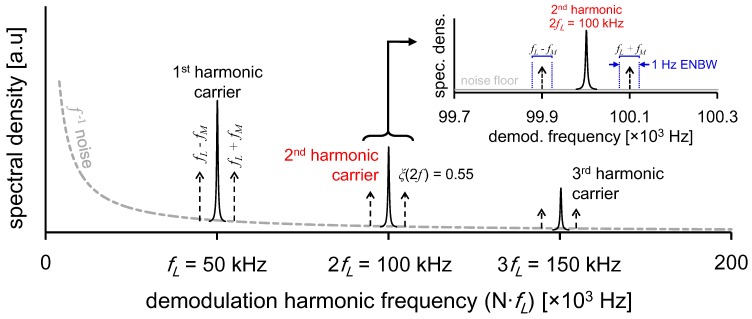
Spectral schematic of DM-FRS signal frequencies. Modulation of the laser occurs at *f_L_* = 50 kHz, resulting in wavelength modulated carrier frequencies at *N*·*f_L_* and a corresponding decrease in 1/*f* noise. Further modulation of the magnetic field (*f_M_ =* 100 Hz) results in generation of sum and difference frequency sidebands *N*·*f_L_* ± *f_M_* (scales in schematic are exaggerated for clarity). In DM-FRS, the demodulation bandwidth Δ*f* must be considered around each sideband, resulting in 2·Δ*f* total noise bandwidth.

An alternative technique (DM-FRS, or dual-modulation Faraday rotation spectroscopy) developed by Wang *et al.* [13] utilizes simultaneous laser wavelength (*f_L_*) and magnetic field (*f_M_*) modulation, thus combining the laser intensity-noise reduction with etalon-free measurements, enabling near shot-noise and baseline free detection. This dual-modulation method encodes the FRS signal into high-frequency sidebands (*N·f_L_* ± *f_M_*) where the appropriate harmonic (*N*) may be chosen for signal demodulation (Figure 1). This DM-FRS approach has consistently yielded detection sensitivities beyond what is experimentally realizable using conventional FRS techniques, achieving record noise-equivalent angles (Θ_NEA_) below 10^−8^ rad·Hz^−1/2^ [13,14]. In the following sections, we rigorously consider the effect of dual-sideband signals and harmonic detection on the fundamental limits of measurement, and analytically compare DM-FRS with the conventional FRS techniques.

### Comparison of DM-FRS to Conventional FRS Methods

In accordance with the above discussion, we find that in practice AC-FRS is limited by laser intensity-noise and DC-FRS by optical etalons, whereas DM-FRS has been designed to address both limitations. However, it is instructive to consider all three techniques operating in ideal conditions and examine their ultimate sensing capabilities in the regime of the fundamental limit.

In the case of the conventional 90°-method (nearly crossed polarizers), the signal (V_90°_) and noise (σ_90°_) is given by [17]:

(1) $$V_{90{}^{\circ}}=R_{v}\cdot R_{i}\cdot P_{0}\cdot \sin(2\alpha )\cdot {\text{Θ}}_{FRS}$$

(2) $${\text{σ}}_{90{}^{\circ}}=\sqrt{\;{\left[R_{v}\cdot R_{i}\cdot \text{RIN}(f_{L,90{}^{\circ}})\cdot P_{sig}\cdot \sqrt{\text{Δ}f}\right]}^{2}+{\left[R_{v}\sqrt{2eR_{i}\cdot P_{sig}}\cdot \sqrt{\text{Δ}f}\right]}^{2}+{\left[R_{v}\cdot R_{i}\cdot \text{NEP}\cdot \sqrt{\text{Δ}f}\right]}^{2}}$$

where the photodetector noise-equivalent power (NEP) and laser relative intensity-noise (RIN) is considered to be frequency dependent. P_0_ is the power incident upon the analyzer, with α and Θ_FRS_ denoting the analyzer uncrossing angle and Faraday rotation angle respectively. The signal power incident on the photodetector is given by P_sig_ = P_0_·sin^2^(α). Δf is defined as the equivalent noise bandwidth (ENBW), related inversely to the minimum time between adjacent data samples ensuring independent (uncorrelated) measurements. Both AC-FRS and DC-FRS can be implemented using the 90°-method, and the above nomenclature can be used for signal and noise analysis (with a distinction of the RIN measured at frequencies that correspond to harmonics of field modulation or laser wavelength modulation in AC-FRS and DC-FRS respectively).

In DM-FRS, magnetic field modulation of the sample produces an amplitude modulation which appears as two sidebands around a carrier frequency associated with fast wavelength modulation of the laser. The sidebands must be separately demodulated and summed to produce the resultant FRS signal. This can be generally understood as a two-step process of signal generation involving the harmonic detection of a wavelength-modulated spectrum of an AC-FRS lineshape [13,14]. Since harmonic detection reduces the maximum available signal by ξ(Nf_L_), and dual-sideband demodulation doubles the bandwidth required for signal retrieval (*i.e*., for ENBW of Δf = 1 Hz, dual sideband detection introduces noise from actual bandwidth of 2·Δf = 2 Hz), these effects are taken into account in signal and noise modeling. We thus have:

(3) $$V_{DM-FRS}=\text{ξ}(Nf_{L})\cdot R_{v}\cdot R_{i}\cdot P_{0}\cdot \sin(2\text{α})\cdot {\text{Θ}}_{FRS}$$

(4) $${\text{σ}}_{DM-FRS}=\sqrt{\begin{array}{l} \;{\left[R_{v}\cdot R_{i}\cdot \text{RIN}(f_{L,DM-FRS})\cdot P_{sig}\cdot \sqrt{2\text{Δ}f}\right]}^{2}+\\ \;{\left[R_{v}\sqrt{2eR_{i}\cdot P_{sig}}\cdot \sqrt{2\text{Δ}f}\right]}^{2}+{\left[R_{v}\cdot R_{i}\cdot \text{NEP}\cdot \sqrt{2\text{Δ}f}\right]}^{2} \end{array}}$$

For fundamental signal-to-noise ratio (SNR) considerations, we may consider the quantum shot-noise limiting case with:

(5) $${\text{σ}}_{90{}^{\circ},\;shot}=R_{v}\cdot \sqrt{2eR_{i}\cdot P_{0}\cdot \sin^{2}(\text{α})}\cdot \sqrt{\text{Δ}f}$$

(6) $${\text{σ}}_{DM-FRS,\;shot}=R_{v}\cdot \sqrt{2eR_{i}\cdot P_{0}\cdot \sin^{2}(\text{α})}\cdot \sqrt{2\text{Δ}f}$$

Thus, the fundamental signal-to-noise (SNR) limit for both measurements becomes (assuming small α):

(7) $$SNR_{90{}^{\circ},\;shot}=\frac{V_{90{}^{\circ}}}{{\text{σ}}_{90{}^{\circ},\;shot}}=\sqrt{\frac{2R_{i}\cdot P_{0}}{e\cdot \text{Δ}f}}\cdot {\text{Θ}}_{FRS}$$

(8) $$SNR_{DM-FRS,\;shot}=\sqrt{\frac{R_{i}\cdot P_{0}}{e\cdot \text{Δ}f}}\cdot {\text{Θ}}_{FRS}\cdot \text{ξ}(Nf_{L})$$

As expected, an additional modulation process in DM-FRS results in additional degradation of the SNR by a factor of $\text{β}\equiv \frac{SNR_{\text{DM-FRS, shot}}}{SNR_{90{}^{\circ}\text{, shot}}}=\frac{\text{ξ}(Nf_{L})}{\sqrt{2}}$ with respect to idealized 90°-methods. In the nominal case of 2nd-harmonic demodulation, ξ(*2f_L_*) of 0.55 is achieved yielding α = 0.39, hence, fundamentally, DM-FRS can achieve ~40% of the detection limit of shot-noise limited AC-FRS techniques. However, the ultimate shot-noise limit is difficult to attain in conventional FRS methods, whereas even though the fundamental limit in Equation (8) is inferior to Equation (7) by the factor α, DM-FRS is able to reach this limit far more easily in practical systems.

It is useful at this point to also clarify the notational meaning of bandwidth normalized values in this paper: we take the normalization unit Hz^−1/2^ to represent the signal demodulation bandwidth about each DM-FRS sideband (Figure 1), which is understood to be the fastest rate at which we are able to resolve signal changes. Although this notation symbolizes only half the noise bandwidth under consideration, figures of merit quoted in Hz^−1/2^ (implying values normalized to 1 Hz ENBW) represent single-sideband ENBW (consistent with AC-FRS notation), and should be understood as having already accounted for the √2× noise increase from the dual-sidebands.

## 3. Spectrometer Design

**Figure 2:**
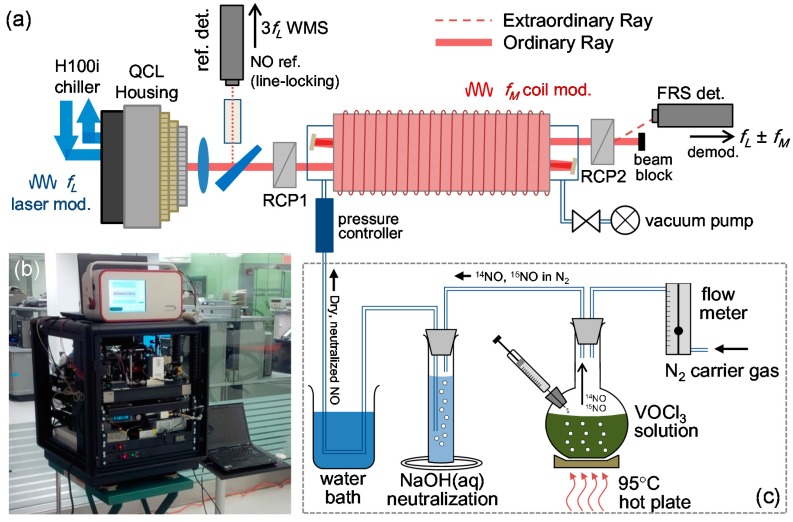
Integrated sensor schematic. (**a**) Dual-modulation Faraday rotation spectrometer for NO isotopic analysis. The system is optimized for minor isotope sensitivity (^15^NO Q(3/2) transition, 1842.763 cm^−1^) with a detection limit of 0.36 ppbv·Hz^−1/2^ using a triple-pass gas cell (45 cm optical pathlength). A reference gas cell (~1% ^15^NO, and ~1% ^14^NO in N_2_) is utilized as wavelength reference for line-locking; (**b**) Photograph of spectrometer housed in 12U 19” rack, integrated with breath sampler; (**c**) Chemical system for nitrate/nitrite conversion to NO via heated acidic vanadium(III).

Following the development of Wang *et al.* [13], measurement of NO is performed by targeting the ^15^N^16^O Q(3/2) transition at 1842.763 cm^−1^ (minor isotope) and ^14^N^16^O P(19/2)e doublet transition at 1842.946 cm^−1^ (major isotope) using a distributed-feedback quantum cascade laser (Alpes Lasers). A Mercury-Cadmium-Telluride photodetector (Vigo Systems, PVI-3TE-6, current responsivity *R_i_* = 3.8 A/W and voltage transimpedance *R_v_* = 4.5 × 10^4^ V/A) was used. Substantial spectrometer size/weight reduction was accomplished by replacement of the water chiller with a fan and coolant-based heat-sink (Corsair H100i), and use of a miniaturized class-D audio amplifier (Sure Electronics TK2050), data acquisition card (NI USB-6001) and vacuum pump (KNF UN84.3 ANDC). Sample measurement is accomplished through the triple pass gas cell (*L_cell_* = 15 cm) operating in continuous flow configuration at 80 Torr and magnetic field *B* = 150 Gauss. The DM-FRS spectrometer (Figure 2) utilizes simultaneous modulation of laser wavelength (*f_L_* = 50 kHz) and magnetic field (*f_M_* = 100 Hz) [15], where the former reduces 1/*f* noise and the latter eliminates optical etalon effects as described in the previous section. The magnetic field modulation frequency was reduced from [15] to lower electromagnetic interference effects, eliminating the need for high-permeability shielding. Retrieval of DM-FRS spectra is performed via demodulation at signal sideband harmonics (2*f_L_* ± *f_M_*), enabling low laser-technical noise, near baseline-free measurement capability, along with intrinsic immunity to interferences from water and other diamagnetic absorbers in the mid-infrared region. A reference branch with a gas cell containing both NO isotopes (~1% ^15^NO, and ~1% ^14^NO in N_2_) is used to perform spectral line-locking of the laser wavelength to the major or minor NO isotope transitions for continuous measurements.

The spectrometer is housed within a 12U 19” rack (Rackmount Solutions 212024-L) and is integrated with a wet-chemistry nitrate/nitrite to NO conversion system for fluid sample analysis (Figure 2c). Gas samples containing NO can be introduced directly to the system for isotopic analysis (Figure 2b also shows a commercial human breath sampler by Loccioni, which can be used with the system for medical studies).

## 4. DM-FRS System Performance

In the following sections system performance analysis is presented in three stages: (i) SNR optimization; (ii) spectrum acquisition and baseline performance; and (iii) measurement stability, accuracy, as well as detection limits for ^15^NO. In Section 4.2 the mechanism newly developed in this system to provide time-multiplexed isotopic analysis via line-switching between the major and minor isotopic transitions is also described.

### 4.1. Signal-to-Noise Ratio Optimization

General optimization of SNR is required for FRS spectrometers due to different contributions of detector-noise (σ*_det_*), laser intensity-noise (σ*_int_*), and shot-noise (σ*_shot_*). These noise contributions are characterized by controlled variation of the analyzer uncrossing angle (α), thereby altering the signal power as *P_sig_* = *P*_0_·sin^2^(α), where *P*_0_ = 6.4 mW is the power incident upon the analyzer. Analysis of the noise yields a laser relative intensity-noise (RIN) of 2.19 × 10^−7^ Hz^−1/2^, and a photodetector noise equivalent power (NEP) of 1.25 × 10^−12^ W·Hz^−1/2^. The optimum uncrossing is well approximated (for small α) by the point where σ*_int_* = σ*_det_* and the effective extinction ratio of the optical system of ε = 1.86 × 10^−4^ [15] (polarizers and optical gas cell) is assumed to have negligible noise contribution, yielding a calculated optimum angle consistent with the measured α*_opt_* = 1.71° at *P_sig_* = 5.7 μW.

### 4.2. DM-FRS Spectrum Acquisition and Time-Multiplexed Isotopic Analysis

**Figure 3:**
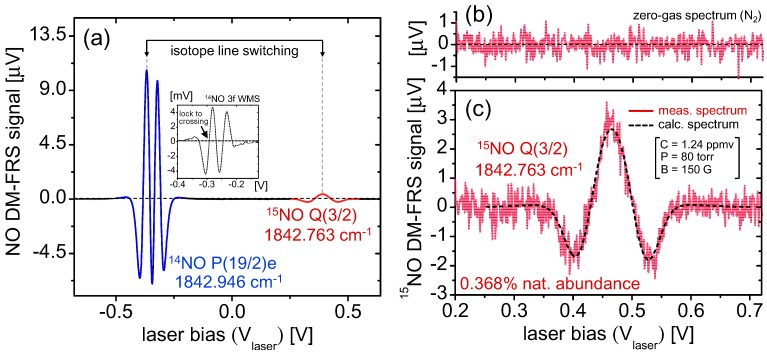
(**a**) Spectra of ^14^NO P(19/2)e (blue) and ^15^NO Q(3/2) (red) transitions from a G-Cal permeation device. Quasi-simultaneous isotopic analysis via line switching can be performed by alternately locking to the 3*f_L_* WMS zero-crossing each isotope; (**b**) Zero-gas spectrum using cylinder nitrogen demonstrating near-zero baseline spectra; (**c**) Measurement of ^15^NO spectra using non-certified NO in N_2_ mixture. Spectral modeling gives ^15^NO concentration of 4.56 ppbv, corresponding to 1.24 ppmv ^14^NO at natural abundance.

Signal spectra for both ^14^NO and ^15^NO are obtained by current-tuning of the laser across both transitions (Figure 3). A G-Cal permeation device (Vici Metronics) was used to generate NO in N_2_ gas sample. The acquired spectrum (Figure 3a) includes both ^14^NO P(19/2)e at 1842.946 cm^−1^ and ^15^NO Q(3/2) at 1842.763cm^−1^ lines (blue and red respectively) in the fundamental *v*_2_ ro-vibrational band of NO. The spectrum was acquired using 1500 points at Δ*f* = 1 Hz ENBW per point, resulting in a total acquisition time of ~1500 s. The inset of Figure 3a shows a corresponding typical 3*f_L_* wavelength modulated absorption spectrum for ^14^NO measured in the reference channel, which was used to line-lock to the maxima of the DM-FRS spectra of the major isotope. Qualitative analysis of line-lock stability yielded a lock-speed of ~2.5 s, whereupon the oscillations of the PID (proportional-integral-derivative) controlled feedback loop resulted in signal oscillations below the detection limit of the system. Within the lock-speed limitations, line-switching (isotopic multiplexing) was performed by hopping the laser current set-point between the ^15^NO and ^14^NO transitions at predefined time intervals for quasi-simultaneous measurements of isotopic variations. It is important to note that the ratio of the raw FRS signals is not directly indicative of the isotopic abundance as system parameters were optimized for maximum ^15^NO sensitivity by targeting the Q(3/2) transition with the highest effective magnetic moment, which despite lower line intensity produces the highest FRS signals at low magnetic fields. Therefore, an appropriate signal calibration must be utilized to measure relative isotopic abundances (see Section 5 for details).

Figure 3b depicts the ^15^NO spectra acquired with pure nitrogen as sample gas, indicating zero-baseline performance near the fundamental shot-noise limit. This demonstrates the benefit of lower magnetic field modulation frequency causing the reduction of EMI without the need for any extraneous shielding material for our laser or detector systems. Figure 3c is the ^15^NO Q(3/2) transition measured for uncalibrated cylinder gas containing ~1–2 ppmv of NO in N_2_ mixture. Along with the measurement is a spectra simulated for the same transition using the model from [23]:

(9) $$H_{2}(\tilde{\text{ν}})=\frac{G}{D}\cdot \frac{2}{\text{π}}\int_{0}^{\text{π}}\left[V_{90{}^{\circ}}(\tilde{\text{ν}}+\text{Δ}\tilde{\text{ν}}\cos\text{θ})\right]\cdot \cos(2\text{θ})\cdot d\text{θ}$$

We have defined ῦ as the wavenumber frequency (cm^−1^) and Δῦ as the modulation depth, which for 2nd harmonic DM-FRS corresponds to 1.2× the full-width half maximum of the AC-FRS spectrum [13]. G is the lock-in amplifier input transimpedance, and the signal reduction factor D accounts for the root-mean square measurement (D = √2) performed by the lock-in during the demodulation process. The spectral fit yields a ^15^NO concentration of 4.56 ppbv. With an assumption of the natural abundance (0.368%) one can calculate the concentration of the ^14^NO to be at a level of 1.24 ppmv, which is consistent with the expected concentration levels in this non-certified gas mixture.

### 4.3. Minimum Detection Limits and Long-Term Stability

**Figure 4:**
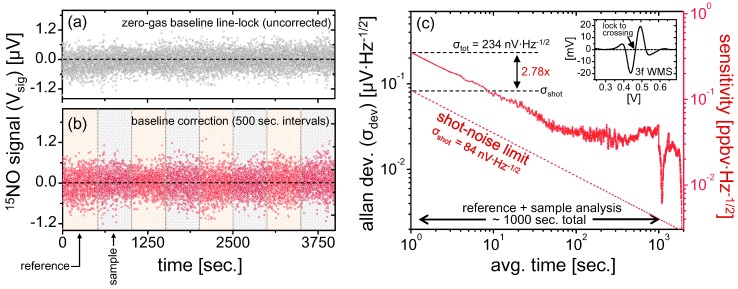
(**a**) Measurement of pure nitrogen by line-locking to the zero-crossing of the ^15^NO 3*f_L_* wavelength modulated spectrum. Slight baseline deviation is visible over the ~1 h of measurement, indicating EMI influence; (**b**) Linear baseline correction every ~500 s is necessary for liquid samples analysis and similar correction is applied here to demonstrate the Allan deviation in a practical measurement scenario; (**c**) Allan deviation of the baseline-corrected measurement, demonstrating white-noise performance up to ~1000 s at 2.8× the shot-noise limit. The inset is a typical 3*f_L_* WMS spectra used for line-locking.

Characterization of the spectrometer stability is performed in a line-locked mode using the ^15^NO transition (Figure 4c, inset shows WMS spectrum used for line-locking). Pure nitrogen is utilized for the test, and all drifts are therefore attributable to instrumental instabilities. The raw measurement data over a span of ~1 h is shown in Figure 4a. A slow positive signal drift is noticeable over this long measurement period, which indicates possible electronic interferences that indirectly modulate optical etalons into FRS signal sidebands. In the case of a practical implementation of the system for measurements of small liquid samples, residual NO produced in the chemical conversion unit must be nulled for each measurement, which requires baseline correction based on the data acquired before and after the sample peak. Typically, sample measurements follow known reference injections, each lasting approximately 500 s at a typical system flow-rate of ~100 sccm. A linear correction for each 500 s measurement interval utilizing the first 50 s and last 100 s is used for baseline correction anyway; therefore to understand instrumental stability in a practical measurement scenario such sample-peak baseline correction technique was applied to data in Figure 4a followed by Allan deviation analysis shown in Figure 4c (corrected data is shown in Figure 4b). As shown in Figure 4c, system precision is set by a noise level of 234 nV·Hz^−1/2^, which is 2.8× the shot-noise limit (σ*_shot_* = 84 nV·Hz^−1/2^). The system is white-noise limited up to 1000 s, which indicates that sequential reference and sample measurements (500 s each) may be performed with linear baseline correction without introducing any substantial drifts in measurement accuracy. Furthermore, the detection limit for ^15^NO is derived using the ratio of ^15^NO signal (Figure 3c) and system noise (Figure 4c): SNR = 3 μV/(234 nV·Hz^−1/2^) = 12.8 Hz^1/2^, resulting in a bandwidth normalized minimum ^15^NO detection limit of 4.56 ppbv/(12.8 Hz^1/2^) = 0.36 ppbv·Hz^−1/2^. This corresponds to a noise-equivalent Faraday rotation angle Θ_NEA_ of [13]:

(10) $${\text{Θ}}_{NEA}=\frac{{\text{σ}}_{tot}}{{\text{σ}}_{shot}}\cdot {\text{Θ}}_{SNEA}=\frac{{\text{σ}}_{tot}}{{\text{σ}}_{shot}}\cdot \frac{1}{\beta }\cdot \sqrt{\frac{h\text{ν}}{2\text{η}P_{0}}}=1.31\times 10^{-8}\;rad\cdot Hz^{-1/2}$$

where Θ_SNEA_ is the shot-noise equivalent Faraday rotation angle (*i.e.*, the rotation angle corresponding to shot-noise fluctuations), and a modification factor 1/α = √2/*ξ*(2*f_L_*) from Equations (7) and (8) is used to account for harmonic signal detection and dual-sideband noise. For comparison to absorption-based spectrometers, we calculate a minimum fractional absorption of *ΔP*/*P*_0_ ≈ (α*L*)_min_ = 6.27 × 10^−8^ Hz^−1/2^, which compares favorably to typical spectroscopic systems which provide Δ*P*/*P_0_* in the ~10^−4^ Hz^−1/2^ range [24].

## 5. Isotopic Ratiometry and Real-Time Fractionation Studies

For fluid sample analysis a full integration of the spectrometer and chemical conversion setup (Figure 2) has been performed. In this Section we demonstrate fractionation-free spectrometer performance in isotopic analysis of gas samples, and measure real-time fractionation during injection of liquid samples together with an error analysis which indicates excellent linearity and consistent sub-permil precision for μmol sample sizes.

### 5.1. Real-Time Analysis of Gas Samples

In Section 4, spectrometer noise characterization revealed a drift-free performance of approximately 1000 s, and it is expected that within this time frame the DM-FRS spectrometer provides high accuracy, and does not introduce measurement artefacts (*i.e.*, modification of the ratiometric value via instrument fractionation). The system measurements that are used to evaluate the isotopic ratios (δ^15^N) are typically presented as permil (‰) values, defined according to [25]:

(11) δ15N = [[N15]S  [N14]S[N15]R  [N14]R-1] × 103 ‰ 

where [N]_S_ and [N]_R_ represent the true sample and reference volume concentrations of NO. In the case of experimentally determined concentrations, we define an analogous ratiometric quantity:

(12) δ15N˜ = [[N˜15]S  [N˜14]S[N˜15]R  [N˜14]R-1] × 103 ‰

We define the corresponding tilde notation to denote the raw measured signal on the spectrometer (typically measured in μV). The equality between volume concentration ratio (ppmv) defined in Equation (11) and raw signal ratio (μV) in Equation (12) generally holds true since [^15^Ñ] = [^15^N]·γ_15_ and [^14^Ñ] = [^14^N]·γ_14_ where γ is the ppmv to μV instrumental conversion factor for each isotope. Assuming sequential reference and sample measurements are made within the drift-free time-frame of the instrument (see Section 4.3), we expect γ to remain the same (*i.e.*, γ_S14_ = γ_R14_ and γ_S15_ = γ_R15_), and therefore we expect δ^15^N = δ^15^Ñ (*i.e.*, the reference ratio to remain unchanged) within the stability time of the spectrometer. Within the preceding stability constraints and also for notational clarity, we use δ^15^N to denote our measured isotopic ratio in the following discussion.

To evaluate the isotopic ratiometry performance of the system, we first consider the instantaneous ratiometric error Δ(δ^15^N)(*t*):

(13) δ15N(t) ± Δ(δ15N)(t)  = [[N˜15]S(t) ± Δ[N˜15]S  [N˜14]S(t) ± Δ[N˜14]S[N˜15]R [N˜14]R - 1] × 103 ‰ 

where we have defined our instantaneous signals [^15^Ñ]_s_(*t*) and [^14^Ñ]_s_(*t*) to be time dependent and our uncertainties Δ[^15^Ñ]_s_ and Δ[^14^Ñ]_s_ represent errors for data reported every 1 s (1 Hz measurements). The reference measurement error is assumed to be negligible, which can be assured through application of high concentration reference gases/liquid samples. By applying isotopic line switching with 50% measurement duty cycle between major and minor isotopes, it is appropriate to assume Δ[^15^Ñ]_s_/[^15^Ñ]_s_ > Δ[^14^Ñ]_s_/[^14^Ñ]_s_. Propagation of the uncertainties in Equation (13) then gives:

(14) Δ(δ15N)(t)= [N˜15]S(t) [N˜14]S(t)[N˜15]R  [N˜14]R(Δ[N˜15]S [N˜15]S(t))2+(Δ[N˜14]S [N˜14]S(t))2 ×103≈ (δ15N(t)103+1)(Δ[N˜15]S [N˜15]S(t))×103

For samples with near-natural abundance, Equation (14) reduces further to a straightforward relation between the ratiometric error and the relative precision of minor isotope detection:

(15) Δ(δ15N)(t)≈(Δ[N˜15]S [N˜15]S(t))×103

From Equations (14) and (15), it is evident that error contributions of the spectrometer are inversely proportional to the minor isotope signal strength [^15^Ñ]_s_(*t*). Thus, any noise and drifts in the system can be compensated by increasing the sample size for isotopic ratiometry measurements. As a rough approximation, with Δ[^15^Ñ]_s_ = 234 nV·Hz^−1/2^ indicated in Figure 4c, a sub-permil precision can be achieved for 1 Hz measurements if ~100 ppmv NO samples are used, which would result in [^15^Ñ]_s_ > 234 μV. With this relationship, one can predict minimum sample size required to achieve desirable instantaneous ratiometric precision.

**Figure 5:**
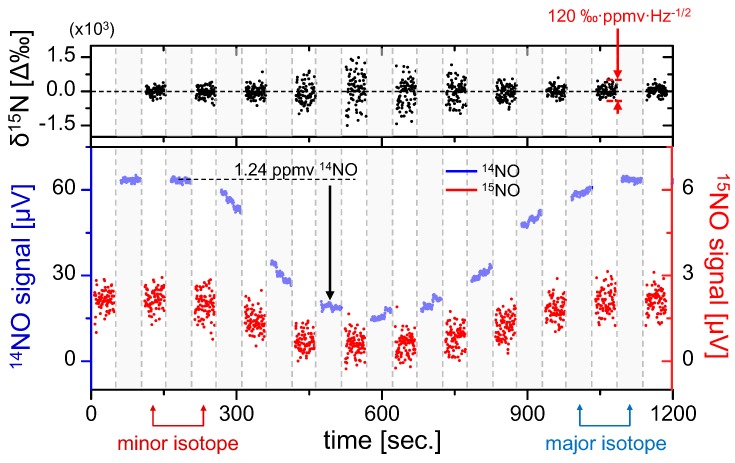
Quasi-simultaneous isotope measurement of 1.24 ppmv ^14^NO in N_2_ mixture diluted using pure nitrogen. Line-switching occurs every 50 s, and polynomial interpolation of ^14^NO is used to calculate the isotopic ratio (top graph). Gray regions denote ^14^NO measurements and white regions denote ^15^NO. Permil ratiometric values are calculated according to Equation (12), demonstrating fractionation-free system performance. A concentration-normalized precision of Δ(δ^15^N) = 120‰·ppmv·Hz^−1/2^ was determined from a typical measurement segment.

Both the ratiometric precision and its concentration dependence as well as any potential fractionation effects of the gas analyzer can be conveniently evaluated using gas samples. To this end, we used the NO in N_2_ mixture from the cylinder (with 1.24 ppmv ^14^NO concentration as calculated in Section 4.2), which was manually diluted over a time span of 1200 s via mixing with dry nitrogen to produce a gradual concentration variation in the sample gas concentration with no modification to the relative isotopic composition of NO. For this test, only relative excursion from the mean isotopic composition is of importance, and the sample was assumed to have natural abundance of NO isotopes with nominal δ^15^N = 0. The line-switch interval was set to 50 s (Figure 5), with the gray shaded regions denoting ^14^NO measurement periods and the non-shaded regions denoting ^15^NO. Inherent to the line-switch process is the presence of gaps in the measurement of each isotope. To enable direct ratiometric calculation in the measurement regions of ^15^NO, we perform a polynomial interpolation of the ^14^NO signal (which has significantly higher SNR and negligible effect on the ratiometric error as shown in Equation (14)).

The results of isotopic analysis are shown in the top graph of Figure 5, demonstrating negligible fractionation effects over the duration of NO variation. It is also clearly visible that the precision is degraded with smaller sample concentrations, which was predicted by Equations (14) and (15). Despite varying precision, during this test statistically the permil ratio does not deviate from the mean (0‰ assumed here), which confirms that the accuracy of the sensor is preserved over the ~1000 s measurement time, a result consistent with the Allan analysis in Figure 4. Within a single 50 s measurement segment we have estimated a concentration-normalized ratiometric precision of 120‰·ppmv·Hz^−1/2^. This confirms the previous estimate predicting that a sub-permil precision for a 1 Hz measurement near natural abundance requires NO concentrations of >100 ppmv.

### 5.2. Fluid Sample Analysis

**Figure 6:**
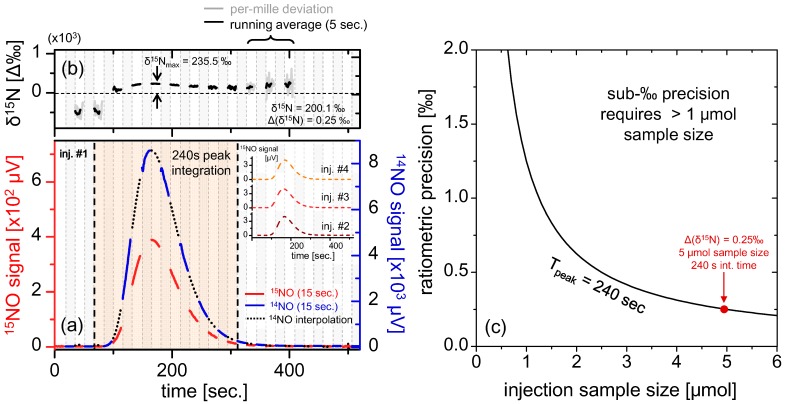
(**a**) Real-time measurement of 5 μmol injection of KNO_3_ (500 μL of 10 mM solution) via line-switching every 15 s at 50% duty cycle for each isotope. The blue, red and black segments correspond to [^14^Ñ]_S_, [^15^Ñ]_S_ and interpolated [^14^Ñ]_S_ respectively. The resulting peak has FWHM of ~100 s, obtained in a flow rate of 64 sccm, and the inset shows three more repeat injections (only [^15^Ñ]_S_ is shown for clarity); (**b**) Ratiometric curves derived from the quotient of [^15^Ñ]_S_(*t*) and interpolated [^14^Ñ]_S_. A clear time dependent curvature is apparent, with a maxima occurring at the measurement peak; (**c**) Calculation of ratiometric precision *vs.* sample size, using data obtained from (**a**). Generally, samples >1 μmol NO_3_^−^ are required to ensure sub-permil precisions over the span of the peak.

To study real-time fractionation effects in the chemical conversion system for liquid sample analysis, it is necessary to preserve high ratiometric precision. Therefore in order to produce NO concentrations above 100 ppmv, we have used >1 μmol samples of liquid nitrate/nitrite solutions. As a demonstration of the quasi-simultaneous isotope measurement of fluid samples, we have injected 5 μmol (500 μL × 10 mM) KNO_3_ (potassium nitrate) into the acidic vanadium(III) for measurement of NO isotopes as shown in Figure 6. Broad peaks of ~100 s full-width half-maximum (FWHM) were obtained at a flow rate of 64 sccm, and line-switching at 15 s intervals was selected to ensure sufficient time resolution during the sample peak. In principle, the measurement duty cycle for each isotope (^14^NO and ^15^NO) should be optimized such that the majority of time is spent on ^15^NO measurement (which has a lower SNR); however, this results in sparse and short ^14^NO segments, which in turn yields lower peak interpolation accuracy. From these experimental considerations we have chosen a simple 50% duty cycle between major/minor isotope switching. As mentioned in Section 4.2, the PID line-lock requires 2.5 s settling time, and such an interval of data at the beginning of each measurement segment has been discarded prior to analyses. The final isotopic ratio (δ^15^N_T_) is referenced to a NO permeation device (G-Cal, Vici Metronics), whose isotopic ratio (B_R_) has been characterized to remain independent of flow rate (tested from 10 sccm to 140 sccm) and concentration, and is therefore suitable as a stable reference for our measurements.

The data in Figure 6b clearly demonstrate a varying instantaneous permil ratio over the sample peak. Provided that the spectrometer itself is fractionation-free as shown in the previous section, we conclude that the fractionation observed during the liquid sample analysis originates from the chemical conversion unit. Mass fractionation may result in shifted ^15^NO and ^14^NO peaks, and the δ^15^N(*t*) at any given point in time may not be an accurate representation of the sample composition. Therefore to reduce the effects of time-dependent fractionation it is critical to measure the entire peak (*i.e.*, integrating the entire peak ensures we have taken all chemically produced NO molecules into account). To calculate the total permil value δ^15^N_T_ the ratio of the integrated peaks is used according to:

(16) δ15NT = [∫T[N˜15]S(t)∫T[N˜14]S(t)⋅1BR-1] × 103 = [〈[N˜15]S(t)〉T〈[N˜14]S(t)〉T⋅1BR-1] × 103

where we define B_R_ as the reference ratio (G-Cal permeation device), and T as the time-span used for ^15^NO measurement (peak integration time is ~50% total peak time due to isotope switching). Using [^15^Ñ]_s_(*t*) = B_R_·[δ^15^N(*t*)/10^3^ + 1]·[^14^Ñ]_s_(*t*) from Equation (12), we obtain

(17) δ15NT = 〈δ15N(t)⋅[N˜14]S(t)〉T〈[N˜14]S(t)〉T

This demonstrates that the final isotopic ratio can be considered as a weighted average of the instantaneous permil ratio (Figure 6b) over the major isotopic peak. Furthermore, Equation (17) indicates that as [^14^Ñ]_s_(*t*) degrades near the wings of the peak, the relative contribution of δ^15^N(*t*) to δ^15^N_T_ becomes proportionally smaller. Qualitatively, the accuracy of the final ratiometric calculation is hardly affected by the integration of the wings, and any residual accuracy deviation is corrected via calibration with known references.

Propagation of error in Equation (17) and noting that the 1 Hz precisions Δ[^15^Ñ]_s_ are uncorrelated and sum in quadrature, will result in a simple extension of Equation (14) of the following form:

(18) Δ(δ15NT) = Δ[N˜15]ST〈[N˜15]S(t)〉T⋅[δ15NT103+1]×103

Intuitively, Equation (18) can be understood as the quotient of time-averaged noise and signal for the minor isotope, multiplied by a conversion factor (~10^3^ for samples near B_R_ abundance) to convert to ratiometric (‰) units. Since in case of the liquid samples the entire sample peak is used for measurement, the time-averaging further improves ratiometric precision, which is also sample size dependent. The effect of sample size variation on ratiometric precision is plotted in Figure 6c, which indicates sub-‰ precision for sample sizes >1 μmol. For the peak shown in Figure 6a derived from a 5 μmol KNO_3_ sample, we calculate δ^15^N_T_ = 200.1‰ and Δ(δ^15^N_T_) = 0.25‰, demonstrating ratiometric precision well within sub-permil range. Multiple repeat injections yielded very similar ratiometric values (three [^15^Ñ]_S_(*t*) measurements are shown in the inset, and [^14^Ñ]_S_(*t*) is excluded for clarity) with the average isotopic ratio of (δ^15^N_T_)_avg_ = 199.0‰. The standard deviation of (δ^15^N_T_)_stdev_ = 5.08‰ was calculated for the best four out of five injections, which is significantly higher than: (i) instrument precision of 0.25‰ (calculated for 5 μmol KNO_3_) and (ii) instrument drift (sub-‰ for ~10^3^ s as calculated from Figure 4c); therefore, we conclude the principal contribution to the measurement error originates from uncertainty introduced by the chemical reduction of nitrates within the sampling front-end.

### 5.3. Ratiometric Linearity

**Figure 7:**
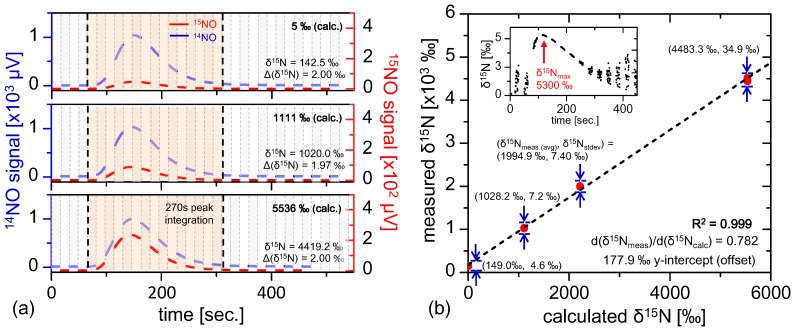
(**a**) Multiple injections of known isotopically labeled references spanning from 5‰ to 5536‰, where the ^15^NO signal is clearly enhanced for enriched samples. The smaller 400 nmol injection size reduces ratiometric precision (Δ(δ^15^N) ≈ 2‰), consistent with Figure 6c and Equation (18); Plotted in (**b**) are multiple labeled reference injections (4 points per calculated δ^15^N value, and the plot demonstrates excellent linearity (R^2^ = 0.999) with measured δ^15^N offset consistent with fractionation observed in Figure 6a. The inset shows the shape of real-time fractionation curve of the most enriched reference injection (δ^15^N_calc_ = 5536‰), demonstrating results similar to those in Section 5.2.

In Section 5.1, fractionation-free system performance was demonstrated, supporting the conclusion that the real-time fractionation described in Section 5.2 may be attributed to isotopic variations in the sample gas. At this point it is appropriate to consider how the ratiometric accuracy varies with isotopic composition. This sensor linearity test was conducted by performing 400 nmol injections of samples with known isotopic compositions with ratiometric values of 5‰, 1111‰, 2218‰, and 5536‰ prepared using a mixture of 99% ^15^N enriched potassium nitrate (Cambridge Isotopes) with natural abundance nitrate (Fisher Scientific). The samples injected were substantially smaller (by roughly 12.5×) than prior injections demonstrated in Section 5.2, thus yielding lower ratiometric precision of Δ(δ^15^N) = 2‰ (consistent with Equation (18) and Figure 6b). Four repeat injections were conducted for each ratiometric sample, and the results are plotted in Figure 7b. The ratiometric analyses demonstrate excellent linearity (R^2^ = 0.999), with a ratiometric offset of 177.9‰ that is consistent with those observed with near-natural abundance samples shown in Figure 6a. Additionally, it is interesting to note that the ratio of measured to calculated isotopic ratio is not unity (dδ^15^N_meas_/dδ^15^N_calc_ = 0.782), indicating possible preference of ^14^NO in the vanadium(III) conversion process. Different temperature dependence of the transition linestrength between ^14^NO and ^15^NO have been considered as a source of this effect, but calculations yield slope modification factors far below the observed deviation for temperature differences up to hundreds of Kelvin. Nevertheless, the excellent linearity demonstrates consistency in the sample conversion and measurement process, thus allowing any sample to be calibrated via known references.

## 6. Conclusions

A dual-modulation Faraday rotation spectrometer is demonstrated for isotopic ratiometry via line-switching. Noise characterization demonstrates 0.36 ppbv·Hz^−1/2 15^NO sensitivity, at 2.8× the shot-noise limit and 1.31 × 10^−9^ rad·Hz^−1/2^ noise-equivalent angle. Ratiometric analysis indicates sub-permil precision for micro-mole level nitrate samples, allowing real-time ratiometric diagnostics with excellent linearity (R^2^ = 0.999) up to 10^3^‰ levels. The combination of sensitivity and transportability demonstrates promise for *in situ* environmental and medical diagnostics. Development of a next-generation portable prototype is underway, with goals of further miniaturization of the signal processing electronics, and integration with a total nitrogen combustion-based conversion unit for measurement of organic nitrogen samples, which is expected to eliminate isotopic uncertainty introduced by the current chemical conversion unit.
